# Cutaneous squamous cell carcinoma-derived extracellular vesicles exert an oncogenic role by activating cancer-associated fibroblasts

**DOI:** 10.1038/s41420-023-01555-2

**Published:** 2023-07-26

**Authors:** Chen Li, Chengxi Sun, Warangkana Lohcharoenkal, Mohamad Moustafa Ali, Pengwei Xing, Wenyi Zheng, André Görgens, Manuela O. Gustafsson, Samir EL Andaloussi, Enikö Sonkoly, Andor Pivarcsi

**Affiliations:** 1 Department of Medical Biochemistry and Microbiology, Uppsala University, Uppsala, Sweden; 2 Dermatology and Venereology, Department of Medical Sciences, Uppsala University, Uppsala, Sweden; 3 Department of Clinical Laboratory, Cheeloo College of Medicine, Shandong University, 250012 Jinan, Shandong China; 4 Unit of Dermatology and Venerology, Department of Medicine, Karolinska Institutet, Stockholm, SE 17176 Sweden; 5 Department of Immunology, Genetics and Pathology, Uppsala University, Uppsala, Sweden; 6 Department of Laboratory Medicine, Clinical Research Center, Karolinska Institutet, Stockholm, Sweden; 7 Institute for Transfusion Medicine, University Hospital Essen, University of Duisburg-Essen, Essen, Germany

**Keywords:** Squamous cell carcinoma, Cancer microenvironment, Extracellular signalling molecules, Tumour heterogeneity

## Abstract

Cutaneous squamous cell carcinoma (cSCC) is a fast-increasing cancer with metastatic potential. Extracellular vesicles (EVs) are small membrane-bound vesicles that play important roles in intercellular communication, particularly in the tumor microenvironment (TME). Here we report that cSCC cells secrete an increased number of EVs relative to normal human epidermal keratinocytes (NHEKs) and that interfering with the capacity of cSCC to secrete EVs inhibits tumor growth in vivo in a xenograft model of human cSCC. Transcriptome analysis of tumor xenografts by RNA-sequencing enabling the simultaneous quantification of both the human and the mouse transcripts revealed that impaired EV-production of cSCC cells prominently altered the phenotype of stromal cells, in particular genes related to extracellular matrix (ECM)-formation and epithelial-mesenchymal transition (EMT). In line with these results, co-culturing of human dermal fibroblasts (HDFs) with cSCC cells, but not with normal keratinocytes in vitro resulted in acquisition of cancer-associated fibroblast (CAF) phenotype. Interestingly, EVs derived from metastatic cSCC cells, but not primary cSCCs or NHEKs, were efficient in converting HDFs to CAFs. Multiplex bead-based flow cytometry assay and mass-spectrometry (MS)-based proteomic analyses revealed the heterogenous cargo of cSCC-derived EVs and that especially EVs derived from metastatic cSCCs carry proteins associated with EV-biogenesis, EMT, and cell migration. Mechanistically, EVs from metastatic cSCC cells result in the activation of TGFβ signaling in HDFs. Altogether, our study suggests that cSCC-derived EVs mediate cancer-stroma communication, in particular the conversion of fibroblasts to CAFs, which eventually contribute to cSCC progression.

## Introduction

Cutaneous squamous cell carcinoma (cSCC) is one of the most prevalent cancers with metastatic potential [1]. Patients with primary cSCCs have a favorable prognosis, as malignant tumors are typically remediable with prompt curative surgeries [2]. Nevertheless, five-year survival drops dramatically to less than 30% for advanced cSCC with metastases [3], making cSCC the second leading cause of skin cancer-related death after melanoma. The key risk factor for cSCC is chronic exposure to ultraviolet (UV)-radiation [4, 5]. Failed repair of UV-induced DNA-damage results in the accumulation of somatic mutations and makes cSCC one of the most highly mutated cancers [6]. As metastasis is the leading cause of mortality in cSCC, investigating the molecular events during cSCC progression is of vital importance for a better understanding of the disease and management of patients with cSCC.

Extracellular vesicle (EV) is the generic term for a highly heterogeneous group of double-layered phospholipid membrane vesicles of different cellular origin, biogenesis, content, and function [7, 8]. EVs can be secreted by almost all cell types and initially were considered as “waste carriers” for eliminating redundant cell components. In recent years, the biological significance of EVs has been well accepted, especially for their functions in mediating intercellular communication through enclosing and transmitting biomolecular cargo (proteins, lipids and nucleic acids) from their parental cells to acceptor cells [9]. Substantial evidence suggests that EVs can participate in tumorigenesis through carrying oncogenic proteins and nucleic acids [10–12]. The tumor microenvironment (TME) is made up of stromal cells (fibroblasts, immune cells, neuroendocrine cells, and adipose cells) and extracellular matrix (ECM) surrounding cancer cells, which exerts a profound effect on the behavior of cancer cells [13, 14]. EVs are critical mediators for establishing intercellular communication between tumor and stromal cells and contribute to a tumor-promoting TME faciliting cancer initiation, progression, and metastasis [15–17].

Cancer-associated fibroblasts (CAFs) constitute a major part of stromal cells in solid tumors, and are responsible for producing cytokines, chemokines, and ECM proteins [18]. CAFs actively participate in cancer development and progression through enhancing proliferation, angiogenesis, immune evasion, and ECM remodeling [19, 20]. Cancer cell-derived EVs have been shown to activate fibroblasts in TME into CAFs, thereby promoting tumor growth and invasion via EV-loaded cargoes-proteins (e.g., TGFβ [21]) or nucleic acids (e.g., miR-10b [22]). However little has been known about their roles in cSCC.

In this study, we report the comprehensive analysis of EVs produced by a panel cSCC cell lines and normal human epidermal keratinocytes (NHEKs). We found that (1) cSCC cells secrete a higher amount of EVs compared to NHEKs; (2) secretion of EVs by the RAB27A-pathway contributes to cSCC tumor growth in vivo. (3) cSCC cell-derived EVs mediate the intercellular communication between cancer-stroma cells and drive the conversion of fibroblasts into CAFs. (4) By analyzing the protein composition of EVs secreted by NHEKs and cSCC cells, we identify a striking heterogeneity regarding surface epitopes and total proteins, among which EVs produced by cancer cells with high metastatic capacity exhibit the most diverse proteins.

## Results

### Increased production of EVs in cSCCs compared to primary keratinocytes

**Fig. 1:**
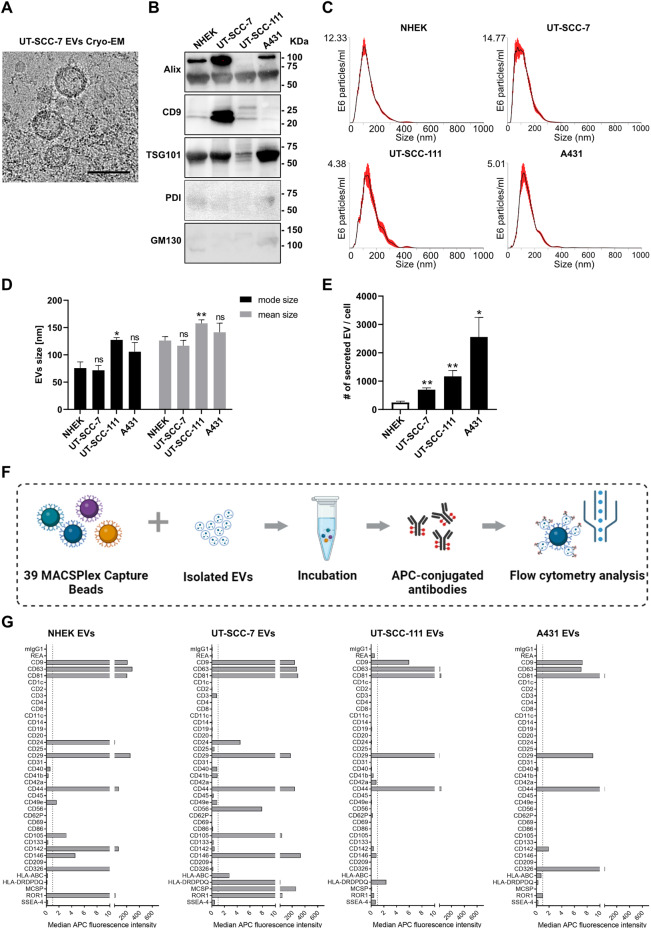
Characterization of EVs produced by normal epidermal keratinocytes and cSCC cell lines. **A** Cryo-electron microscopy analysis of EVs produced by UT-SCC-7 cSCC cells. Scale bar = 100 nm. **B** Extracellular vesicles were obtained from NHEK, UT-SCC-7, UT-SCC-111, and A431 cSCC cell lines and lysates were analyzed by immunoblotting assay. **C** Nanoparticle tracking analysis (NTA) showing the size distribution of EVs released from NHEK and cSCC cell lines UT-SCC-7, UT-SCC-111, and A431. Red error bars indicate mean ± SEM. **D** Comparison of the size distribution between EVs released from NHEK (*n* = 4) and cSCC cell lines UT-SCC-7 (*n* = 4), UT-SCC-111 (*n* = 3) and A431 (*n* = 3). *P < 0.05, **P < 0.01, n.s. not significant, one-way ANOVA. **E** The number of EVs produced by NHEK (*n* = 4) and cSCC cell lines UT-SCC-7 (*n* = 3), UT-SCC-111 (*n* = 3) and A431 (*n* = 3) on a per-cell basis. *P < 0.05, **P < 0.01, one-way ANOVA. Data are presented as mean ± SEM. **F** Schematic of multiplex bead-based flow cytometry analysis of isolated EVs. MACSPlex Capture Beads with 37 surface epitopes and two isotype controls were used, followed by incubating with APC-conjugated antibodies against tetraspanins CD9, CD63, and CD81 and analyzed by flow cytometry. Created using BioRender (BioRender.com). **G** The level of 37 surface proteins were analyzed on EVs obtained from NHEK and cSCC cell lines UT-SCC7, UT-SCC-111, and A431 by a multiplex bead-based flow-cytometry assay.

EVs secreted by normal human epidermal keratinocytes (NHEKs) and three cSCC cell lines - two from primary (UT-SCC-111 and A431) and one from metastatic tumor (UT-SCC-7) - were collected from conditioned medium (CM) by size-exclusion chromatography. The presence and morphology of EVs were visualized by transmission electron microscopy (TEM) which showed a typical cup-shape vesicular structure (Supplementary Fig. 1A). High-resolution EV images were acquired by cryogenic electron microscopy (cryo-EM), which showed EVs as round double-layered membranous vesicles (Fig. 1A). The commonly used EV markers Alix, CD9 and TSG101 were detected in EV samples from both NHEKs and cSCC cell lines by immunoblotting while PDI (Endoplasmic reticulum marker) and GM130 (Golgi marker) were undetectable (Fig. 1B). Further confirming the ability of the method to isolate EVs, tetraspanin positive (CD9, CD63 or CD81) EVs were detected by Imaging Flow Cytometry (IFCM) analysis from all samples (Supplementary Fig. 1B, C). The size and concentration of EVs were measured by nanoparticle tracking analysis (NTA) which suggested the mean particle diameter of our EVs sample was mainly ranging from 110 to 160 nm (Fig. 1C, D). EVs produced by cSCC cell line UT-SCC-111 appeared with larger size than EVs from NHEK. Otherwise, the cellular origin of EVs could not be determined based on their size-distribution (Fig. 1D). To evaluate the ability of different cells to produce EVs, the number of EVs produced on a per-cell-basis was calculated (Fig. 1E) by normalizing NTA-results to cell counts. We found that cSCC cell lines secreted more EVs than NHEKs, with A431 cells producing the highest number of EVs on a per cell basis (Fig. 1E).

Next, we aimed to determine the surface composition of EVs produced by NHEKs and cSCC cell lines. To this end, a total of 37 surface proteins were detected by a multiplex bead-based flow cytometry assay in EV samples isolated from the conditioned supernatants of NHEKs and cSCC cell lines UT-SCC-7, UT-SCC-111, and A431 (Fig. 1F). As expected, EVs positive for tetraspanin CD9, CD63, or CD81 were detected in all EVs samples (Fig. 1G and Supplementary Fig. 1D). Remarkably, several surface molecules (CD56, CD105, major histocompatibility complex (MHC) class I and II molecules and MCSP) which are associated with cancer development were detected exclusively on EVs produced by the metastatic cSCC cell line, UT-SCC-7 (Supplementary Fig. 1D). These results showed that the production and/or release of EVs is elevated in cSCC cells compared with normal keratinocytes and that there is a substantial heterogeneity among metastatic cSCC and primary cSCCs in terms of their EV-surface proteins.

### Inhibition of EV-production in cSCC cells interferes with primary tumor growth in vivo

**Fig. 2:**
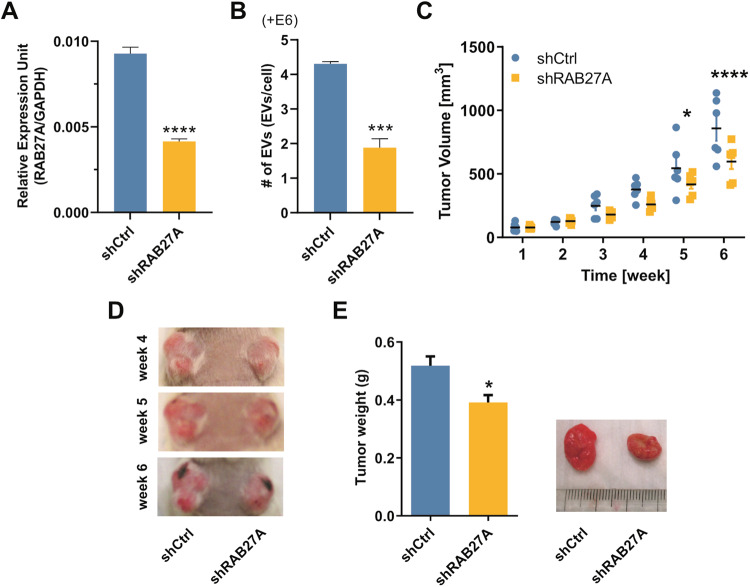
Inhibition of EV-production interferes with primary cSCC tumor growth in vivo. **A** The efficiency of shRNA-mediated knockdown of RAB27A in UT-SCC-7 cells was determined by qRT-PCR. ****P < 0.0001, Student’s *t* test, *n* = 4. **B** Production of EVs by control or RAB27 shRNA-transduced UT-SCC-7 cells was analyzed by NTA. ***P < 0.001, Student’s *t* test, *n* = 3. **C** Primary tumor growth was monitored after the subcutaneous injection of control (shCtrl) and RAB27A-knockdown (shRAB27A) UT-SCC-7 cells into immunocompromised mice. *P < 0.05, ****P < 0.0001. Two-way ANOVA. Six immunocompromised mice were used for xenograft models. **D** Representative images of tumors formed on mice at week 4, 5, and 6. **E** Weight of tumors formed by control and RAB27A-knockdown UT-SCC-7 cell lines was measured after the termination of the experiment at week 6. *P < 0.05, Wilcoxon matched-pairs sighed rank test, 6 xenografts per group. Data are presented as mean ± SEM.

The level of circulating tumor-derived EVs is associated with tumor progression [23, 24]. The fact that cSCC cells secrete more EVs than normal keratinocytes prompted us to investigate whether they could contribute to tumor growth. To answer this question, we established a stable RAB27A-knockdown UT-SCC-7 cell line using shRNA-mediated lentiviral transduction (Fig. 2A). RAB27A (Ras-related protein Rab-27A) is a pivotal protein that controls EV-secretion through modulating the docking of the multivesicular body (MVB) to the plasma membrane [25]. As expected, stable depletion of RAB27A in UT-SCC-7 cells significantly reduced the amount of secreted EVs as determined by NTA (Fig. 2B).

To study the effect of cSCC cell-derived EVs on tumor growth, xenograft models were established by subcutaneously injecting control and RAB27A knockdown UT-SCC-7 cells into immunocompromised mice. Through monitoring tumor growth weekly, we observed that the volume of tumors formed by RAB27A-depleted UT-SCC-7 cells was smaller than those formed by control cells (Fig. 2C, D). Consistently, RAB27A-knockdown in UT-SCC-7 cells inhibited the growth of tumor weight (Fig. 2E). Taken together, our data reveal that EV-secretion inhibition in cSCC cells resulted from RAB27A-depletion impaired tumor growth in vivo.

### cSCC cells regulate surrounding stromal cell gene expression

**Fig. 3:**
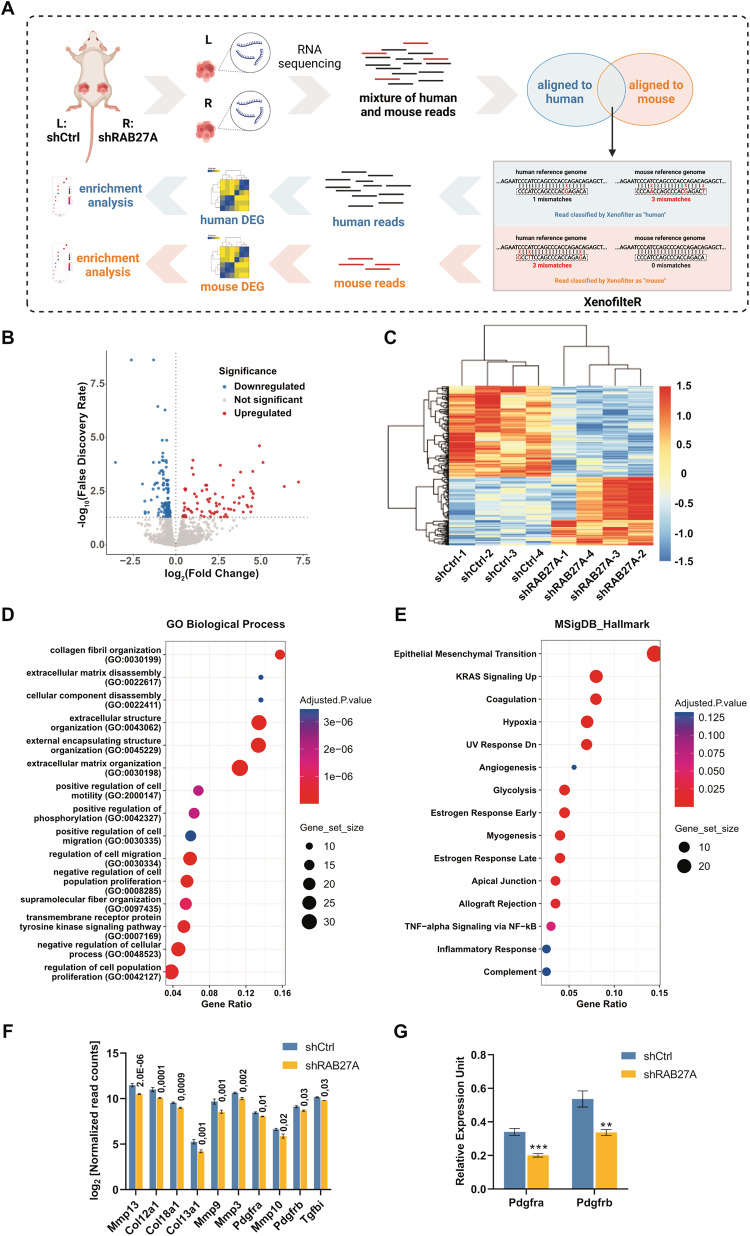
Inhibition of EV production in cSCC cells results in altered transcriptomic profiles of stromal cells in tumor xenografts. **A** Schematic representation of the transcriptome analysis of cSCC tumor xenografts. RNA from tumor xenografts, representing a mixture of cSCC cell-derived (i.e. human) and stromal cell-derived (i.e. mouse) transcripts, was extracted. After RNA-sequencing, unique human sequence reads and unique mouse reads were separated through alignment to the human (GRCh38) and mouse (GRCm39) reference genomes. Reads with partial match to both the human and mouse genome were further separated based on the edit-distance between a sequence read and reference genome using the XenofilteR-pipeline. Thereafter, differential expression and enrichment analyses were performed on human and mouse transcripts separately. Figure was created using BioRender (BioRender.com). **B** Volcano-plot showing differential expression of mouse mRNAs between stroma cells of shRAB27A and shCtrl xenografts. Vertical lines denote the fold change cutoff, while the horizontal line denotes the FDR cutoff. Red color represents upregulated and blue color represents downregulated coding transcripts. **C** Heatmap and hierarchical clustering of differentially expressed mRNAs in stromal cells (FDR < 0.05). **D**, **E** Top 15 significantly enriched pathways among genes downregulated in the stroma of RAB27A-knockdown xenograft tumors (FDR < 0.05): **D** GO Biological Processes 2021 and **E** MSigDB Hallmarks. The color of the nodes indicates the adjusted *P*-value and the size of the nodes reflects the number of overlapping genes in the gene sets. **F** Log_2_-normalized read counts of ten representative downregulated mouse genes in the ‘extracellular matrix organization’ (GO:0030198) and ‘regulation of cell migration’ (GO:0030334) pathways. FDRs were shown. **G** The expression level of Pdgfra and Pdgfrb in xenografts were detected by qRT-PCR. **P < 0.01, ***P < 0.001, Student’s *t* test, 6 xenografts per group. Data are presented as mean ± SEM.

The observation that RAB27A-depletion suppressed tumor growth in vivo made us hypothesize that cSCC cell-derived EVs could promote tumor growth through educating stromal cells in TME and shaping a tumor-supportive microenvironment. To gain further insight into the effect of cSCC cell-derived EVs on cells in the tumor stroma, we performed a whole transcriptome analysis on xenograft tumors (Fig. 3A). Through parallel alignment of sequencing reads against both the human and the mouse genomes followed by species-specific quantification, we could reconstruct the transcriptome of both the murine (majorly representing tumor-infiltrating stromal cells) and the human cells (representing cSCC cells) in the complex tumor tissue (Fig. 3A) and analyze the consequence of impaired EV-production on the stromal cells, separately. A total of 369 mRNAs were identified to be differentially expressed (FDR < 0.05) in stromal cells of shRAB27-depleted tumors (Fig. 3B, C). Interestingly, genes whose expression was suppressed in stromal cells in the RAB27A-knockdown-group were significantly enriched in pathways associated with extracellular matrix (ECM) organization, epithelial-mesenchymal transition (EMT), cell migration and angiogenesis (Fig. 3D, E and Supplementary Fig. 2A, B). Of note, among genes enriched in GO terms ECM organization and regulation of cell migration pathways, a number of important CAF marker genes (Pdgfra and Pdgfrb) were identified (Fig. 3F). Decreased expression levels of Pdgfra and Pdgfrb in shRAB27A-KD mouse stromal cells were further validated by qRT-PCR (Fig. 3G), suggesting that cSCC cell-secreted EVs may regulate the conversion of normal fibroblasts into CAFs in vivo.

Parallel analysis of transcriptomic changes in the cSCC tumor cells identified a total of 4042 differentially expressed mRNAs (FDR < 0.05) (Supplementary Fig. 3A, B). Genes upregulated in shRAB27A-KD tumors were enriched in pathways related to the p53-pathway and apoptosis (Supplementary Fig. 3C).

Taken together, these data suggested that inhibition of EV-secretion has a profound effect on the transcriptome of cells in the tumor stroma and that it may impair cSCC tumor growth in vivo by regulating stromal cell functions involved in the organization of ECM.

### cSCC cell-derived EVs drive the onset of cancer-associated fibroblast phenotype

**Fig. 4:**
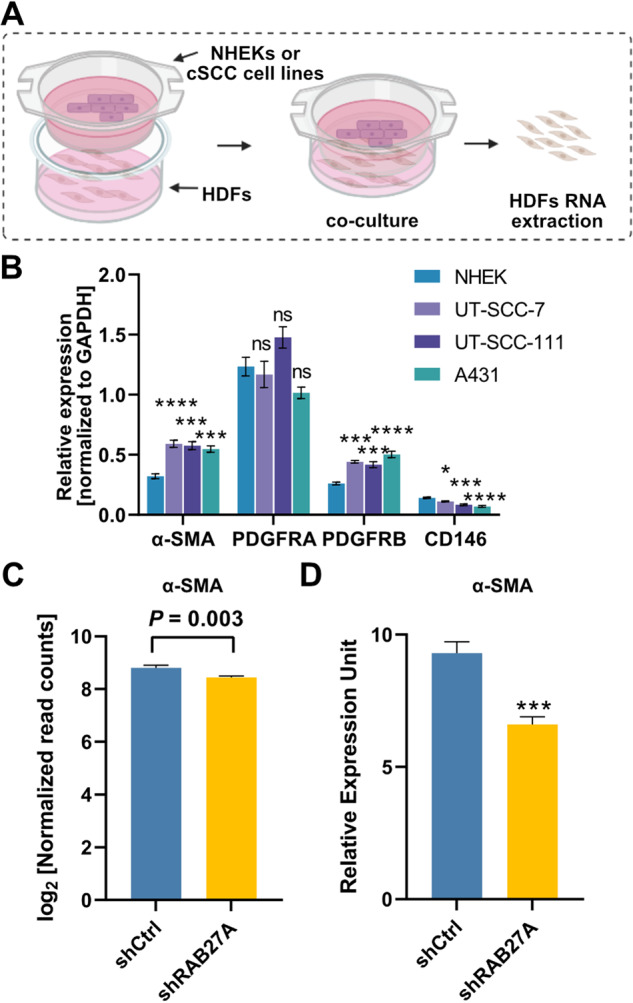
cSCC cells stimulate the transformation of fibroblasts to cancer-associated fibroblasts via soluble mediators. **A** Schematic representation of the co-culture assay of human dermal fibroblasts (HDFs) and NHEK or cSCC cells. Created using BioRender (BioRender.com). **B** Cancer-associated fibroblast (CAF)-related genes were detected in HDFs co-cultured with NHEK or cSCC cells by qRT-PCR. *P < 0.05, **P < 0.01, ***P < 0.001, ****P < 0.0001, n.s. not significant, one-way ANOVA, *n* = 4. **C**, **D** Expression of α-SMA in mouse stromal cells in shRAB27A vs. shCtrl cSCC xenografts was measured by RNAseq (log_2_ -normalized read counts, C) and qRT-PCR analysis (D). ***P < 0.001, Student’s *t*-test, 6 xenografts per group. Data are presented as mean ± SEM.

Growing evidence indicate that EVs are key mediators of intercellular communication in the TME and regulate the formation of CAFs and remodeling of the ECM [26–28]. To investigate the possible communication between cSCC cells and stromal cells, we next co-cultured cSCC cells (from primary tumors: UT-SCC-111 or A431, metastatic cSCC: UT-SCC-7) or primary human keratinocytes (NHEKs) with human dermal fibroblasts (HDFs) (Fig. 4A). The expression of alpha-smooth muscle actin (α-SMA), a marker for CAFs, was increased in HDFs co-cultured with cSCC cells compared with those co-cultured with NHEKs (Fig. 4B). Consistently, decreased expression of α-SMA was detected in mouse stroma cells by RNA-sequencing upon knockdown of RAB27A and impairment of EV-secretion (Fig. 4C) which was also validated by qRT-PCR (Fig. 4D). Moreover, HDFs showed increased expression level of PDGFRB upon co-culturing with cSCC cells, although there was no significant change on expression level of PDGFRA (Fig. 4B), both of which were decreased in mouse stroma upon knockdown of RAB27A. In addition, co-culturing with cSCC cells decreased the expression level of CD146 in HDFs, which is a marker of tumor-suppressive CAFs (Fig. 4B).

**Fig. 5:**
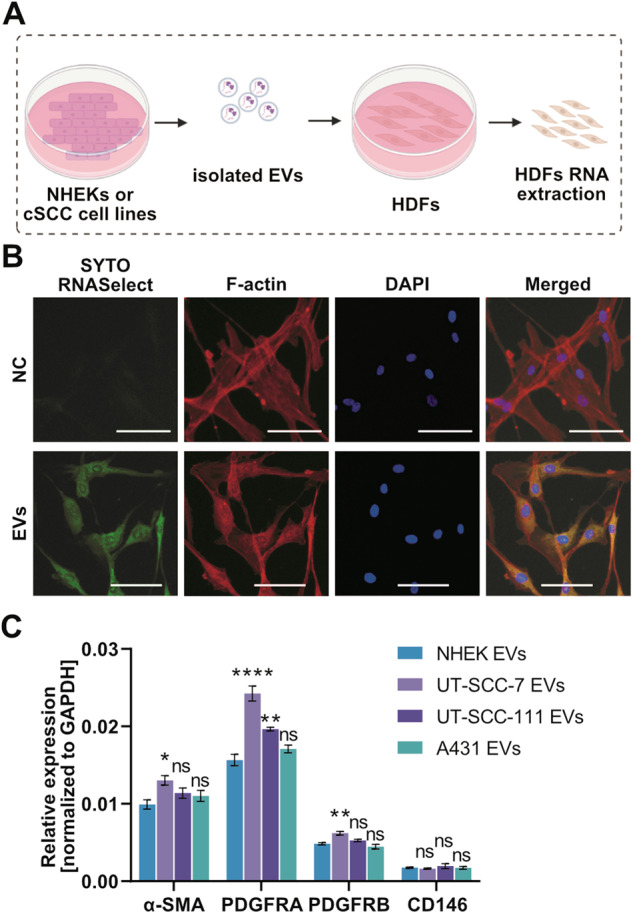
cSCC cell-derived EVs are taken up by fibroblasts and drive the onset of cancer-associated fibroblast phenotypes. **A** Schematic representation of the treatment of HDFs with NHEK- or cSCC cell-derived EVs (corresponding to 20 µg of protein). Created using BioRender (BioRender.com). **B** Uptake of cSCC cell-derived EVs by HDFs was visualized by labeling the EV-cargo (RNA) with SYTO RNASelect green fluorophore. NC: HDFs cultured without EVs added; EVs: HDFs incubated with UT-SCC-7-derived, fluorescently labeled EVs. F-actin was stained with phalloidin, DNA was stained with DAPI. Scale bar = 100 µm. **C** The expression of CAF-marker genes was analyzed by qRT-PCR in HDFs treated with EVs (corresponding to 20 µg of protein) produced by NHEK or cSCC cell lines. *P < 0.05, **P < 0.01, ****P < 0.0001, n.s. not significant, one-way ANOVA, *n* = 4. Data are presented as mean ± SEM.

To further clarify the effects of cSCC cell-derived EVs on HDFs, EVs (corresponding to 20 µg of protein) from NEHK or cSCC cells were used to treat HDFs (Fig. 5A). Uptake of EVs by HDFs was visualized by labeling EVs with SYTO RNASelect green fluorophore (Fig. 5B). Intriguingly, EVs secreted by the different cSCC cells used in our study showed varying ability on fibroblasts education: EVs secreted by metastatic UT-SCC-7 cells were efficient to induce CAF-onset, as measured by the increased expression level of α-SMA, PDGFRA, and PDGFRB (Fig. 5C). UT-SCC-111 cell-derived EVs could only induce the expression of PDGFRA (Fig. 5C), while A431 cell-derived EVs had no obvious effect on the expression level of any of these CAF-related genes (Fig. 5C).

These results suggest that cSCC-derived EVs can regulate the acquisition of CAF phenotype.

### EVs from metastatic cSCC cell line UT-SCC-7 carry proteins regulating EMT and TGFβ signaling

**Fig. 6:**
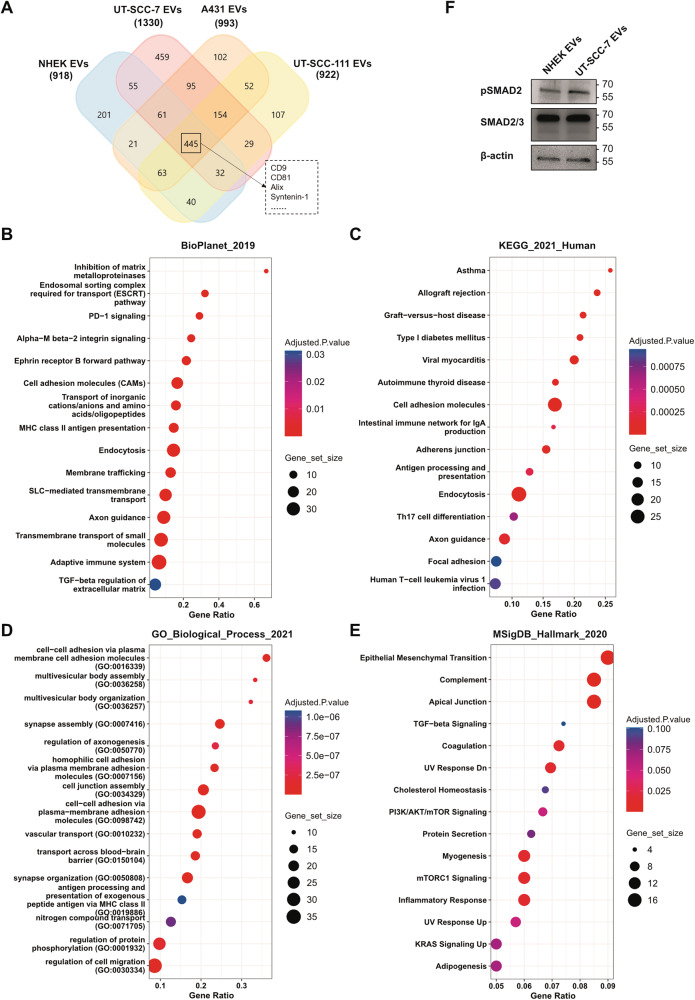
UT-SCC-7 cell-derived EVs carry proteins regulating EVs biogenesis and transportation. **A** Venn-diagram showing the overlap of protein composition of NHEK- and cSCC-derived EVs identified by mass-spectrometry. **B**–**E** Enrichment analysis was performed on proteins expressed exclusively by UT-SCC-7 derived-EVs. Selected significantly enriched pathways from different pathway databases are shown: **B** BioPlanet_2019, **C** KEGG_2021, **D** GO Biological Process 2021, and **E** MSigDB Hallmark 2020. **F** Expression level of phosphorylated and total SMAD2 in HDF incubated with EVs derived from NHEK or UT-SCC-7 cells were measured by western blot. β-actin was used as loading control.

To determine the protein composition of EVs secreted by NHEKs and cSCC cell lines and to identify potential cargo associated with UT-SCC-7-derived EVs, mass-spectrometry (MS) based proteomics analysis was performed. The majority of proteins identified in our EV-samples were consistent with those in the Vesiclepedia database (Vesiclepedia version 4.1 released 15/08/2018, http://www.microvesicles.org/) (Supplementary Fig. 4A) [29, 30]. As expected, proteins associated with exosomes were significantly enriched in EVs isolated from both NHEKs and cSCC cells (Supplementary Fig. 4B–E) [31–33]. In accordance with western-blot and multiplex bead-based profiling experiments, several proteins were commonly found in EVs secreted by both normal and cancerous skin cells, including CD9, C81, Alix, and Synterin-1 (Fig. 6A). Intriguingly, and confirming the results of the multiplex bead-based flow cytometry assay (Fig. 1G and Supplementary Fig. 1D), metastatic cSCC cell line (UT-SCC-7) had the most diverse EV protein-cargo (1330 proteins) compared with EVs secreted by NHEKs and the other two cSCC cell lines (Fig. 6A). To obtain insights into the functions of metastatic UT-SCC-7 cell-derived EVs, enrichment analysis was performed on the list of proteins exclusively present in UT-SCC-7-derived EVs. Interestingly, the proteins exclusively expressed by UT-SCC-7-derived EVs were enriched in functions related to apical junction, EMT, and TGFβ-signaling, an inducer of EMT (Fig. 6B–E), meanwhile several biological processes associated with EV biogenesis were enriched, such as MVB organization, membrane trafficking and vascular transport (Fig. 6B–E). Confirming the biological significance of these findings, the level of phosphorylated SMAD2 was increased in HDFs upon incubation with UT-SCC-7 cell-derived EVs but not with incubation with NEHK-derived EVs (Fig. 6F).

Taken together, efficient EV-production and enhanced EMT may promote cSCC metastasis and cancer progression.

## Discussion

**Fig. 7:**
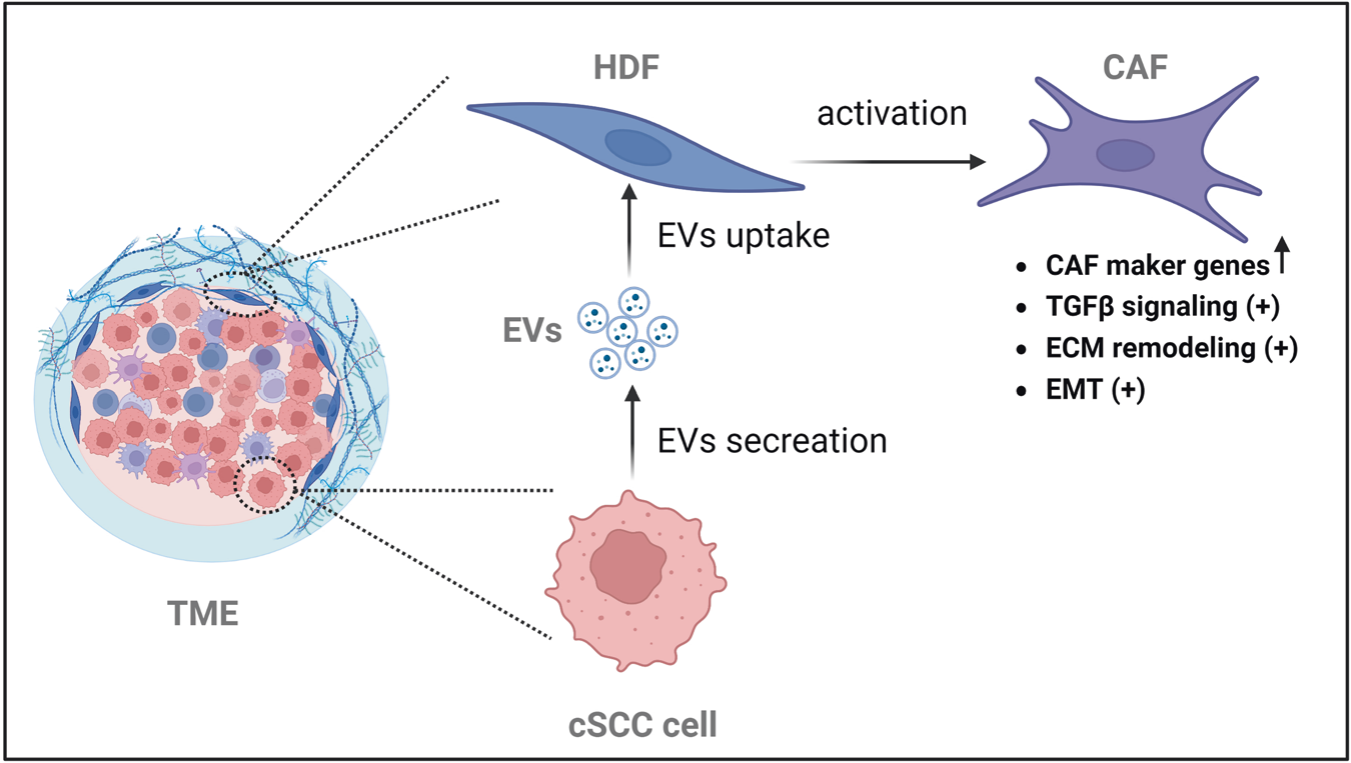
Working hypothesis about the role of cSCC cell-derived EVs on CAF activation in the tumor stroma. Tumor cells in cSCC communicate with fibroblasts and possible other cell types in the tumor microenvironment (TME) via secretion of EVs carrying proteins involved in TGFβ-signaling and epithelial-mesenchymal transition (EMT). EVs may facilitate the acquisition of cancer-associated phenotype (CAF), characterized by enhanced TGFβ signaling, which in turn can support tumor growth. Created using BioRender (BioRender.com).

In this study, we identify an increased production of EVs in cSCC cells compared with normal keratinocytes and show that cancer-derived EVs facilitated cSCC progression in vivo. We demonstrate that cSCC-derived EVs mediated cancer-stroma cell communication in vivo and educated fibroblasts into CAFs in vitro. Interestingly, the capacity of EVs to educate CAFs was variable among cSCC cell lines and metastatic cell line-derived EVs were found to be the most effective in inducing CAF-phenotype (Fig. 7).

In our study, we found that cSCC cell lines produce more EVs compared with normal cells, which is in line with earlier findings obtained with other types of cancers, which have been shown to secrete increased amount of EVs with altered composition relative to their nonmalignant counterparts [34]. However, the driving force for cancer cells producing more EVs has yet to be elucidated. Accumulating evidence suggests that oncogenes and tumor suppressors govern EVs secretion [34], for example, the mutation and/or hyperactivation of EGFR [35–37] and RAS [38, 39]. Of relevance to this, the MAPK pathway is one of the most commonly activated pathways in cSCC [40] due to EGFR-overexpression [41] or mutations in RAS-family members [42, 43] which provide a plausible explanation for our observations about increased EV-production.

To study the effect of cancer-derived EVs on cSCC growth, we inhibited EV-secretion by depletion of RAB27A, a protein controlling EV-secretion. Our xenograft model revealed that in vivo, cSCC tumor growth was significantly impaired by RAB27A-depletion, suggesting that cancer cell-derived EVs are critical for tumor growth. Similar to our findings, RAB27A RNA interference decreased the production of EVs, which further prevented bone marrow education toward a pro-metastatic phenotype, reduced tumor growth and metastasis in melanoma [44]. Interestingly, RAB27A-mediated EVs-secretion is associated with decreased melanoma patient survival [45]. Moreover, RAB27A has been shown to regulate the secretion of EVs in liver cancer stem cells and to contributes to the maintenance of stem-like phenotype and regorafenib resistance [46].

The inhibition of RAB27A-mediated secretion of EVs interfered with cSCC tumor growth in vivo suggesting a role for EVs in the tumor-stroma-communication in cSCC. Indeed, our transcriptome analysis of xenograft tumors revealed an enrichment of genes in ECM-organization among the downregulated genes in stromal cells in the TME. Moreoever, our in vitro co-culture assay indicated that cSCC cells could efficiently activate HDFs to CAFs through paracrine signaling. Incubation of HDFs with EVs confirmed that transformation of CAFs could be driven by metastatic cSCC cell-derived EVs, whereas EVs from non-metastatic cSCC cell lines were not able to transform CAFs. Accumulating evidence reveals that cancer-cell-derived EVs promote the activation of CAFs, which in turn remodel TME to make it more favorable for cancer progression. For example, breast cancer cell-derived EVs promote the activation of CAFs via the transfer of miRNA-125b and contribute to tumor growth [47]. Melanoma‐derived EVs transfer the HSP90/p-IKKα/β complex and activate the IKK/IκB/NF-κB/CXCL1 axis in CAFs and promote angiogenesis [48].

As one of the most highly mutated cancers, cSCC bears a mean somatic mutation rate of 50 mutations per megabase pair DNA, corresponding to an average of ca. 1700 mutations per tumor exome [6, 49, 50]. The intricate mutational landscape is reflected by the enormous phenotypic and functional heterogeneity of cSCCs. Consistently, we observed a huge heterogeneity in terms of EV-protein composition (both surface molecules and protein cargoes) by surface signature analysis and proteomics by MS. To address the potential mechanisms of action of EVs secreted by cSCC cells on driving the onset of CAFs, we were primarily interested in those proteins that were present at a high level on metastatic cSCC cell line-derived EVs. Interestingly, several surface molecules exclusively expressed on metastatic UT-SCC-7 cell-derived EVs were associated with cancer progression, for example, CD105 (endoglin), which has a crucial role in angiogenesis and its elevated level is correlated with tumor metastasis [51]. Elevated expression level of CD56 (neural cell adhesion molecule; NCAM1) has been detected in a range of cancers and it is associated with the diagnosis or prognosis of small cell lung cancer [52], as well as Merkel cell-carcinoma [53] and ovarian cancer [54]. Our proteomic analysis identified several hundred proteins exclusively present in UT-SCC-7 cell-derived EVs enriched in pathways involved in EV biogenesis and uptake, as well as EMT induction and response. Interestingly, metastasis-promoting tetraspanin CD151, which has been reported to support metastatic settlement and matrix remodeling as EV cargo [55], was detected exclusively in UT-SCC-7 cell-derived EVs. Another protein exclusively expressed in UT-SCC-7 cell-derived EVs was Desmoglein 2 (Dsg2). It has been reported that Dsg2-expressing SCC cells produced more EVs, formed larger xenograft tumors, and Dsg2 was shown to facilitate the transfer of EVs from keratinocytes to fibroblasts [56]. Although limited access to cell lines from metastatic cSCCs is a limitation of our study, the extensive ability of metastatic cSCC cells UT-SCC-7-derived EVs to activate CAFs might be explained by their specific protein cargoes which contribute to efficient EV-mediated intercellular communication and cancer-promoting behaviors.

Taken together, our results suggest that EVs produced by SCCs play a role in the growth of cSCC in vivo through mediating cancer-stroma communication with specific protein cargoes. Our study provides novel insights regarding the understanding of cSCC progression.

## Materials and methods

### Cell culture and transduction

NHEKs from adult donor (Thermo Fisher Scientific, Uppsala, Sweden) were cultured in EpiLife™ Medium (Thermo Fisher Scientific) supplemented with Human Keratinocyte Growth Supplement (HKGS) (Thermo Fisher Scientific), penicillin (50 units/mL), and streptomycin (50 µg/mL) (Thermo Fisher Scientific). HDFs from neonatal donor (Thermo Fisher Scientific) were cultured in Human Fibroblast Expansion Basal Medium (Thermo Fisher Scientific) supplemented with Low Serum Growth Supplement (LSGS) (Thermo Fisher Scientific), penicillin (50 units/mL) and streptomycin (50 µg/mL) (Thermo Fisher Scientific). Human squamous cell carcinoma cell lines UT-SCC-7 (established from metastasis of cSCC) [57] and UT-SCC-111 (established from primary cSCC) [57] (kind gifts from Professor Veli-Matti Kähäri, University of Turku, Finland) were maintained in DMEM (Thermo Fisher Scientific) containing 10% (v/v) heat-inactivated fetal bovine serum (Thermo Fisher Scientific), penicillin (50 units/mL) and streptomycin (50 µg/mL) (Thermo Fisher Scientific), 10 mM HEPES (Thermo Fisher Scientific), and 1X MEM Non-Essential Amino Acids Solution (Thermo Fisher Scientific). Primary cSCC cell line A431 (American Type Culture Collection, ATCC) was cultured in DMEM (Thermo Fisher Scientific, Uppsala, Sweden) containing 10% (v/v) heat-inactivated fetal bovine serum (Thermo Fisher Scientific), penicillin (50 units/mL), and streptomycin (50 µg/mL) (Thermo Fisher Scientific). Heat-inactivated fetal bovine serum was replaced by exosome-depleted Fetal Bovine Serum (Thermo Fisher Scientific) 48 hours before collecting CM for EVs isolation to avoid contamination. Cells were maintained in a humidified incubator at 37 °C and 5% CO_2_.

Stable RAB27A knockdown and control shRNA-expressing UT-SCC-7 cell lines were established by lentiviral transduction using TurboRFP as a selection marker (GeneCopoeia). The transduction was performed according to the manufacturer’s procedure instructions in the presence of 8 µg/ml polybrene (Santa Cruz Biotechnology) The transduced cells were selected based on the expression of RFP within the cells using fluorescent-activated cell sorter (FACS).

### Isolation of EVs from CM by size-exclusion chromatography

EVs in CM were isolated using qEV10 / 35 nm size exclusion columns (IZON, Lyon, France) according to the manufacturer’s instructions. Briefly, cells, cell debris, and larger vesicles were removed by serial centrifugations at 300 × *g* at 4 °C for 10 min, 2000 × *g* at 4 °C for 10 min. CM samples were filtered through a 0.22 μm membrane. CM was concentrated using Amicon® Ultra-15 centrifugal filter devices (Merck, Stockholm, Sweden). Flush the qEV10 / 35 nm size exclusion columns with 90 ml PBS. Cell supernatant was loaded and the 20 ml void volume was immediately collected. The following 20 ml EVs samples after void-volume was collected. EVs samples were concentrated with Amicon® Ultra-15 centrifugal filter devices (Merck). Final EV preparations were stored in PBS supplemented with human albumin and trehalose (PBS-HAT) buffer at -80 °C until usage [58].

### Cryo-electron microscopy analysis

For cryo-EM, a 3 µl aliquot of EV suspension was applied onto the carbon side of glow-discharged (30 s, 20 mA) EM grids, blotted for 3 s at 4 °C with a relative humidity level of 95% and plunge-frozen into the precooled liquid ethane with Mark IV Vitrobot (Thermo Fisher). Frozen grids were transferred onto a A 200 kV Glacios electron microscope mounted with a Falcon III direct electron detector (Thermo Fisher).

### Nanoparticle tracking analysis

To determine the size distribution and concentration of EVs, NTA was performed as described previously [59]. Samples were diluted to an appropriate concentration with 0.22 μm filtered PBS before analysis. NanoSight LM10 instrument (Malvern, UK) equipped with a 405 nm LM12 module and EMCCD camera (DL-658-OEM-630, Andor Technology) was used for NAT. Video acquisition was performed using NTA software v3.2, using a camera level of 12.

### Western blot

Total protein from cells or EVs was extracted using RIPA Lysis and Extraction Buffer (Thermo Fisher Scientific) supplemented with cOmplete™ Protease Inhibitor Cocktail (Sigma-Aldrich, Stockholm, Sweden) and PhosSTOP™ (Sigma-Aldrich). Protein concentration was measured by BCA (Thermo Fisher Scientific). After denaturing (98 °C for 10 min), 20 ng protein per well along with protein ladder (Bio-Rad, Stockholm, Sweden) was loaded for sodium dodecyl sulfate-polyacrylamide gel electrophoresis (SDS-PAGE). Subsequently, proteins were transferred onto nitrocellulose membranes. Nitrocellulose membranes were blocked with 5% milk for 1 hour and incubated with the appropriate primary antibodies at 4 °C overnight with gentle shaking. Membranes were washed thrice with PBST for 10 min and incubated with horseradish peroxidase-coupled isotype-specific secondary antibodies (Dako, Glostrup, Denmark) for 1 h at room temperature. The proteins of interest were visualized using an ECL kit (Thermo Fisher Scientific) and images were developed by ChemiDoc MP Imaging System (Bio-Rad). Primary antibodies for CD9 (#13174), PDI (#3501), and GM130 (#12480) were from Cell Signaling Technology (Cell Signaling Technology, Leiden, The Netherlands). Primary antibodies for Alix (sc-53540) and TSG101 (sc-7964) were purchased from Santa Cruz Biotechnology (Santa Cruz Biotechnology, Heidelberg, Germany). Primary antibody for pSMAD2 (ab280888) and SMAD2/3 (ab202445) were purchased from Abcam (Abcam, Cambridge, UK). β-actin (Abcam, ab8226) was used as control for equal loading and transfer of cell total protein.

### Single-EV imaging flow cytometry analysis

Tetraspanin-positive EVs were quantified by single vesicle Imaging Flow Cytometry (IFCM) on an Amnis Cellstream instrument (Luminex) equipped with 405, 488, 561, and 642 nm lasers) based on previously optimized settings and protocols established on an Amnis Imagestream X MkII instrument [60] and as described previously [58]. In brief, a volume of 25 µL from EV aliquots at a concentration of 5 × 10^9^ NTA-based particles/mL were incubated with a mixture of APC-labeled anti-CD9 (Miltenyi Biotech, clone SN4), anti-CD63 (Miltenyi Biotec, clone H5C6) and anti-CD81 antibodies (Beckman Coulter, clone JS64) at a concentration of 8 nM over-night and diluted 2000-fold in PBS-HAT buffer before data acquisition. Samples were measured from 96-well V bottom multiwell plates (Thermo Fisher Scientific) by using the plate reader of the Cellstream instrument with FSC turned off, SSC laser set to 40%, and all other lasers set to 100%. EVs were defined as SSC (low) by using neonGFP-tagged EVs as biological reference material, and region to quantify APC+ fluorescent events were set according to unstained non-fluorescent samples and single fluorescence positive mNG-tagged reference EV controls as described before [60]. Samples were acquired for 5 minutes at a flow rate of 3.66 µL/min (setting: slow) with CellStream software version 1.2.3 and analyzed with FlowJo Software version 10.5.3 (FlowJo, LLC).

### RNA extraction and qRT-PCR

Total RNA was extracted using Trizol reagent (Thermo Fisher Scientific) according to the manufacturer’s instructions. For analysis of mRNA expression, 250–500 ng of RNA was reverse-transcribed into cDNA with the RevertAid first strand cDNA synthesis kit (Thermo Fisher Scientific). Quantitative realtime PCR analysis was then performed on 10 ng of cDNA using iTaq™ Universal SYBR® Green Supermix (Bio-Rad) on the CFX Opus 96 Real-Time PCR System (Bio-Rad).

### Tumor xenograft experiment

A total of 6 NOD SCID gamma (NSG) mice (Jackson Laboratory) were used for xenograft experiments. After 7 days of acclimatization, 10^7^ of control or stable RAB27A knockdown UT-SCC-7 cells were subcutaneously injected into left and right flank of each mouse, respectively. Cell suspension in 100 µl of ice-cold medium was mixed at 1:1 volume ratio with Matrigel right before the injection. Mice were monitored twice a week for tumor size and general body condition. Tumor width and length were measured by vernier caliper and tumor volume was calculated as (width^2^ × length)/2. When tumor volume reached 1000 mm^3^ or mice conditions reached the humane endpoint, tumors were harvested and weighed.

### Xenograft tumor RNA sequencing

Snap frozen xenograft tissues were homogenized using TissueLyser LT (Qiagen, Kista, Sweden) followed by RNA extraction using the miRNeasey mini kit (Qiagen). RNA quality control was conducted by Bioanalyzer and samples with RNA integrity number (RIN) ≥ 8 were proceeded to library construction using the TruSeq stranded mRNA library preparation kit with polyA selection (Illumina Inc.). Paired-end sequencing employing a read length of 100 base pairs was performed using the NovaSeq 6000 system.

### Bioinformatic analysis of RNA-sequencing

RNA subjected to RNA-sequencing was extracted from tumor xenografts, which contained a mixture of transcripts derived from human cSCC cells and mouse stromal cells. To obtain transcriptomic information for both cell types, the sequence reads were aligned to the human reference genome (GRCh38) and the mouse reference genome (GRCm39). Given the presence of sequence reads exhibiting partial alignment to both the human and mouse genomes, the XenofilteR-pipeline [61] was employed for the deconvolution of mouse and human reads. By comparing the alignment results, unique sequence reads specific to human transcripts and unique sequence reads specific to mouse transcripts were identified. For sequence reads showing partial matches to both genomes, the XenofilteR-pipeline assessed the edit-distance between each read and the reference genomes. This evaluation determined whether the read was more closely related to the human or mouse genome, enabling accurate categorization by resolving ambiguous alignments.

Once the human and mouse sequence reads were separated, subsequent differential gene expression analyses were performed separately for each group using R Bioconductor with RStudio (Version: 2023.03.0+386) and EdgeR package (Version: 3.42.4). To identify pathways and biological processes that were associated with dysregulated human and mouse transcript sets, enrichment analyses were conducted using the interactive gene set enrichment analysis web server, Enrichr (https://maayanlab.cloud/Enrichr/).

### Co-culture assays

For co-culture assays, 24 mm Transwell® with 0.4 µm Pore Polycarbonate Membrane Inserts (Corning Life Science, Tewksbury, MA) were used. Before co-culturing, warm PBS was applied to the interior of the inserts (1 mL) and bottom (1 mL) of wells to allow rehydration for 30 min in humidified tissue culture incubator, 37°C, 5% CO_2_ atmosphere. 6 × 10^4^ of NHEKs, or cSCC cells, were then seeded to the upper inserts with HDFs growing in the bottom six-well plate. Exosome-depleted Fetal Bovine Serum (Thermo Fisher Scientific) was used to maintain cSCC cell lines in order to eliminate possible contamination of EVs from the bovine serum. HDFs were harvested for RNA-extraction after a 48 hours of co-culturing.

### EV tracing assay

EVs were labeled using SYTO RNASelect (Thermo Fisher Scientific) according to the “In vitro labeling of exosome RNA and/or membrane components” protocol. Briefly, SYTO RNA select was added to 100 µl of EV sample to obtain a final dye concentration of 10 µM and incubated at 37 °C for 20 min protected from light. Excess unincorporated dye was removed from the labeled exosomes using Exosome Spin Columns (MW 3,000) (Thermo Fisher Scientific) following the standard protocol. Fluorescently labeled EVs were added to the recipient cells and incubated at 37 °C, for 3 hours. Cells were fixated using 4% paraformaldehyde at room temperature for 20 min and permeabilized using 0.1% Triton® X-100 at room temperature for 3–5 min. F-actin was stained using Alexa Fluor phalloidin (Thermo Fisher Scientific) for 20 min, and cell nuclei were stained with DAPI (Thermo Fisher Scientific) for 5 min at room temperature.

### Multiplex bead-based flow cytometry analysis

EV concentration was determined by NTA and flow cytometric bead-based multiplex EV analysis (MACSPlex Exosome Kit, human, Miltenyi Biotec) was performed as described previously [57]. Briefly, a total of 1 × 10^9^ EVs were diluted with MACSPlex buffer (MPB) to a volume of 120 μl and 15 μl MACSPlex Exosome Capture Beads were added. Filter plate was incubated at room temperature overnight on an orbital shaker protected from light. Beads were washed with 200 μl MPB and resuspended with 135 μl MPB. Then, 5 μl of each MACSPlex Exosome Detection Reagent for CD9, CD63, and CD81 (Miltenyi Biotec) were added and plates were incubated at room temperature for 1 hour on an orbital shaker protected from light. Beads were then washed twice with 200 μl MPB, resuspended in 150 μl MPB, and transferred to V-bottom 96-well plates for flow cytometric analysis on a MACSQuant 10 flow cytometer. Data were analyzed and visualized with FlowJo Software version 10.5.3 (FlowJo, LLC).

### Statistical analysis

Statistical analyses were performed using GraphPad Prism 9. The one-way ANOVA, two-way ANOVA, or two-sided Student’s *t* test were used for specific data. *P* < 0.05 was considered as significant. The variance was compared using an *F* test. Data are presented as mean ± SEM. SEM was calculated based on the independent experiments (*n* = number for independent experiments) as reported in the figure legend. In vitro functional experiments were replicated at least three times in the laboratory.

## Supplementary information

Supplementary information

Western blot_uncropped blots

## Data Availability

RNA-sequencing data that supports the findings of this study have been deposited in the Gene Expression Omnibus under the accession code: GSE230706. All other data supporting the findings of this study are available from the corresponding author.
