# Supplementary material for: Cutaneous squamous cell carcinoma-derived extracellular vesicles exert an oncogenic role by activating cancer-associated fibroblasts
Source: Cell Death Discov. 2023 Jul 26;9:260. doi: 10.1038/s41420-023-01555-2 (PMC10372068; doi:10.1038/s41420-023-01555-2)
Supplement: Supplementary file 1 — Supplementary information [file 41420_2023_1555_MOESM1_ESM.docx]

**Supplementary information**

**Cutaneous squamous cell carcinoma-derived extracellular vesicles exert an oncogenic role by activating cancer-associated fibroblasts**

Chen Li^1,2^, Chengxi Sun^1,3^, Warangkana Lohcharoenkal^4^, Mohamad Moustafa Ali^1^, Pengwei Xing^5^, Wenyi Zheng^6^, André Görgens^6-7^, Manuela O. Gustafsson^6^, Samir EL Andaloussi^6^, Enikö Sonkoly^2,4^ and Andor Pivarcsi^1,2,4^*

*****Correspondence: [andor.pivarcsi@imbim.uu.se](mailto:andor.pivarcsi@imbim.uu.se)

^1^Department of Medical Biochemistry and Microbiology, Uppsala University, Uppsala, Sweden; ^2^Dermatology and Venereology, Department of Medical Sciences, Uppsala University; ^3^Department of Clinical Laboratory, Cheeloo College of Medicine, Shandong University, Jinan 250012, Shandong, China; ^4^Unit of Dermatology and Venerology, Department of Medicine, Karolinska Institutet, Stockholm SE-17176, Sweden; ^5^Department of Immunology, Genetics and Pathology, Uppsala University, Uppsala, Sweden; ^6^Department of Laboratory Medicine, Clinical Research Center, Karolinska Institutet, Stockholm, Sweden; ^7^Institute for Transfusion Medicine, University Hospital Essen, University of Duisburg-Essen, Essen, Germany.

**Supplementary Fig. 1: Characterization of EVs produced by normal epidermal keratinocytes and cSCC cell lines.**

**Supplementary Fig. 2: Knockdown of RAB27A results in transcriptomic changes in stromal cells in tumor xenografts.**

**Supplementary Fig. 3: Transcriptome analysis of human cSCC cells in xenograft tumors.**

## Supplementary Fig. 4: A large proportion of protein cargo in NHEK- and cSCC cell-derived EVs is proteins associated with exosomes.

**Supplementary Figures**


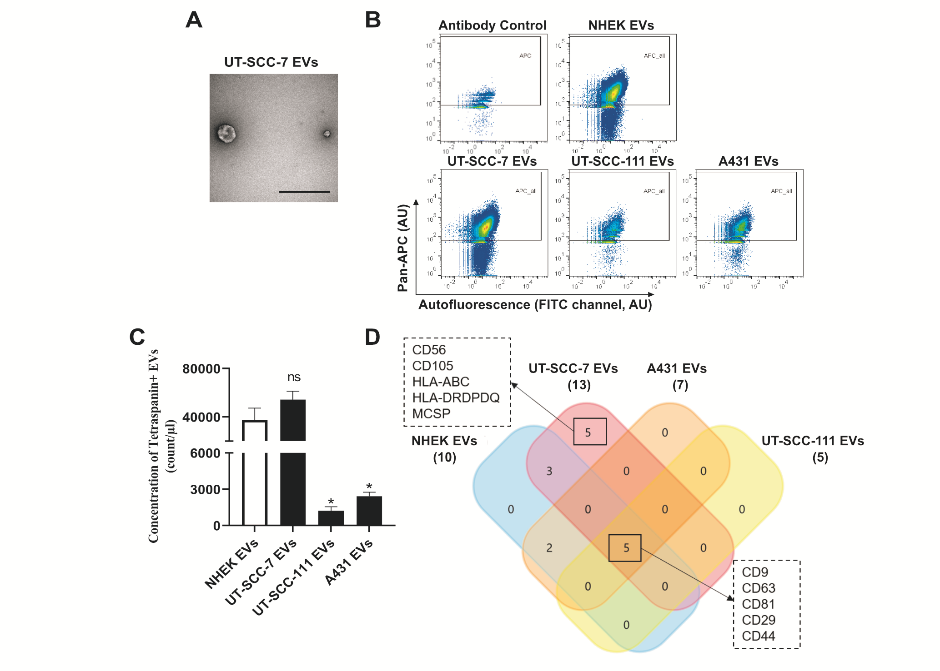


## Supplementary Fig. 1: Characterization of EVs produced by normal epidermal keratinocytes and cSCC cell lines. A Transmission electron microscopy image of EVs produced by UT-SCC-7 cSCC cells. Scale bar = 500 nm. B Imaging Flow Cytometry analysis of tetraspanin positive (CD9, CD63 or CD81) EVs secreted by NHEK, UT-SCC-7, UT-SCC-111 and A431 cSCC cell lines. Antibody Control was conducted without EVs loading. C Quantification of tetraspanin positive EVs released from NHEK (n = 4) and cSCC cell lines UT-SCC-7 (n = 3), UT-SCC-111 (n = 3) and A431 (n = 3) by Cell Stream analysis. **P* < 0.05, n.s. not significant, Student’s *t*-test. Data are presented as mean ± SEM. D Venn-diagram showing the overlap of EV surface markers expressed on EVs derived from NHEK and cSCC cells.

##
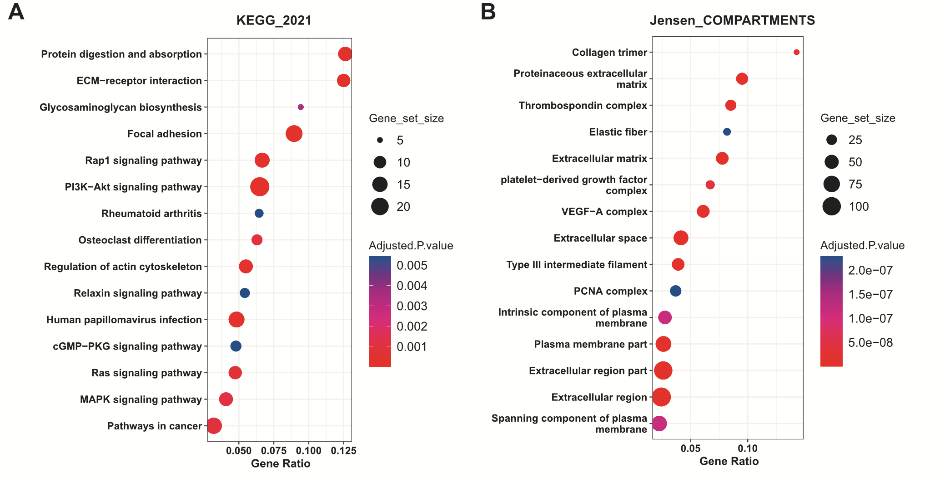


## Supplementary Fig. 2: Knockdown of RAB27A results in transcriptomic changes in stromal cells in tumor xenografts. A-B Top 15 pathways significantly enriched by downregulated mouse genes in RAB27A knockdown xenograft tumors in different pathway databases (FDR < 0.05): (A) KEGG_2021 and (B) Jensen_COMPARTMENTS. The color of the nodes indicates the adjusted p-value and the size of the nodes reflects the number of genes in the gene sets.


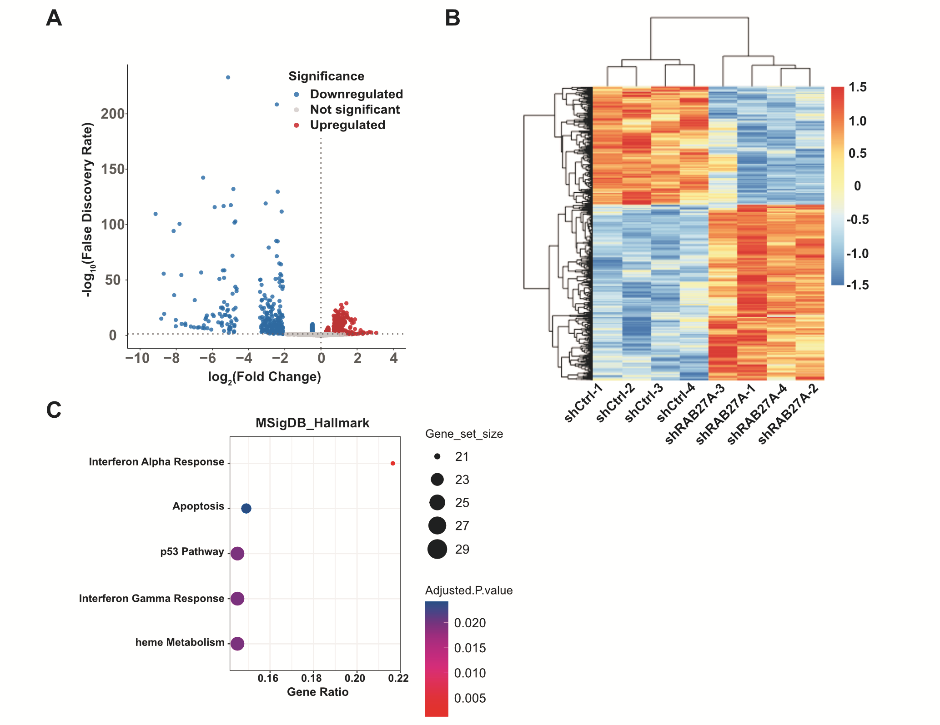


**Supplementary Fig. 3:** **Transcriptome analysis of human cSCC cells in xenograft tumors. A** Volcano plot shows human-derived differentially expressed protein-coding genes in xenograft tissues. Vertical lines denote the fold change cutoff, while the horizontal line denotes the FDR cutoff. Red color represents upregulated and blue color represents downregulated coding transcripts. **B** Heatmap and hierarchical clustering of human-derived differentially expressed protein-coding genes (FDR < 0.05). **C** Top 5 pathways significantly enriched by upregulated human genes in RAB27A knockdown xenografts in MSigDB_Hallmark database (FDR < 0.05). The color of the nodes indicates the adjusted *P*-value and the size of the nodes reflects the number of genes in the gene sets.
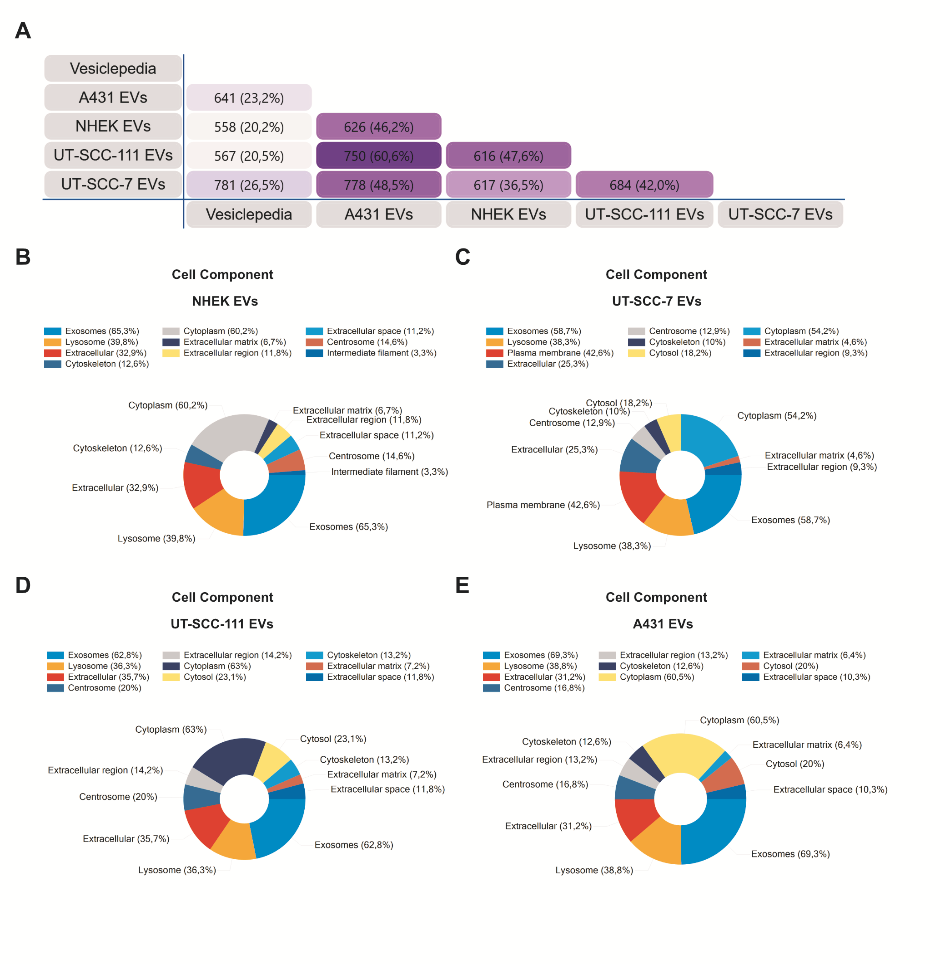


## Supplementary Fig. 4: A large proportion of protein cargo in NHEK- and cSCC cell-derived EVs is proteins associated with exosomes. A Overlap of protein composition of NHEK- and cSCC cells-derived EVs with Vesiclepedia database. B-E Top 10 most significantly enriched pathways from cell component enrichment analysis of proteins expressed by (B) NEHK-, (C) UT-SCC-7-, (D) UT-SCC-111 and (E) A431 derived-EVs.
