# Supplementary material for: Cutaneous squamous cell carcinoma-derived extracellular vesicles exert an oncogenic role by activating cancer-associated fibroblasts
Source: Cell Death Discov. 2023 Jul 26;9:260. doi: 10.1038/s41420-023-01555-2 (PMC10372068; doi:10.1038/s41420-023-01555-2)

Origin data of western blotting assays

Figure 1B

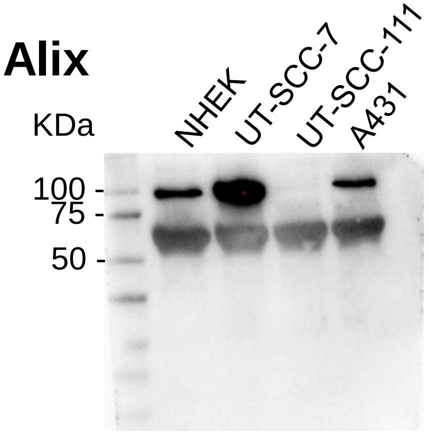

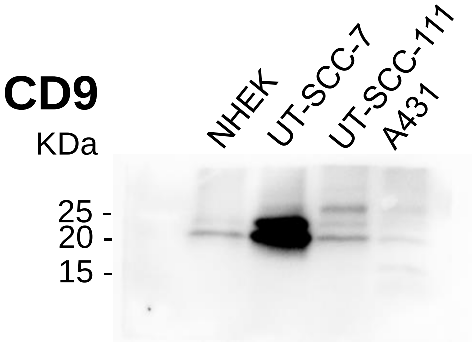

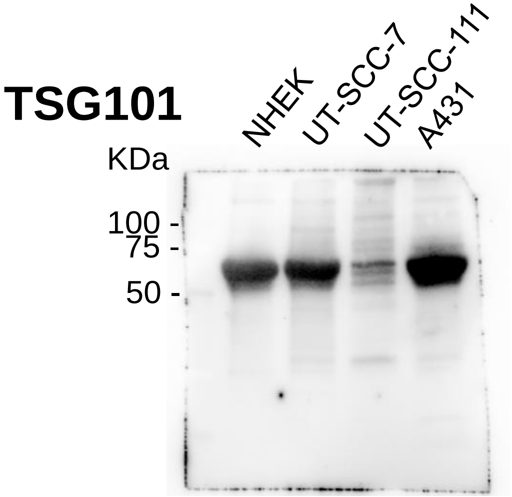

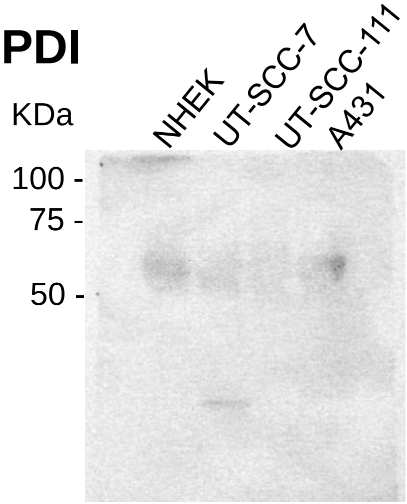

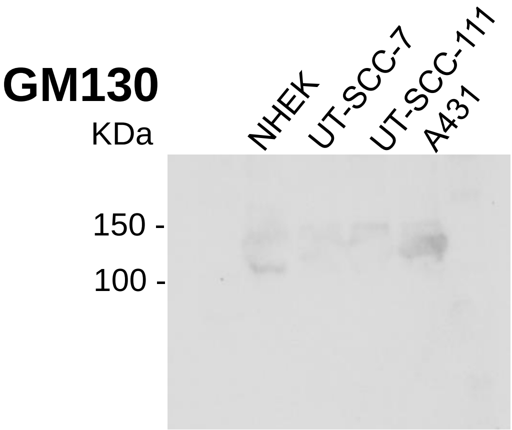

Origin data of western blotting assays

Figure 7F

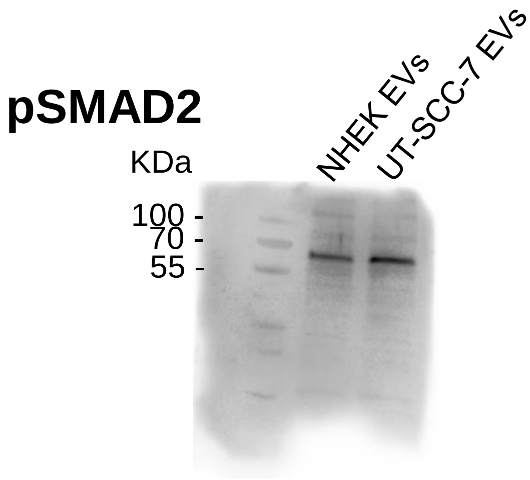

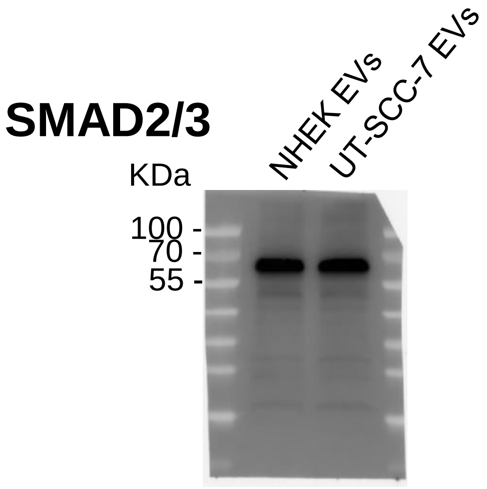

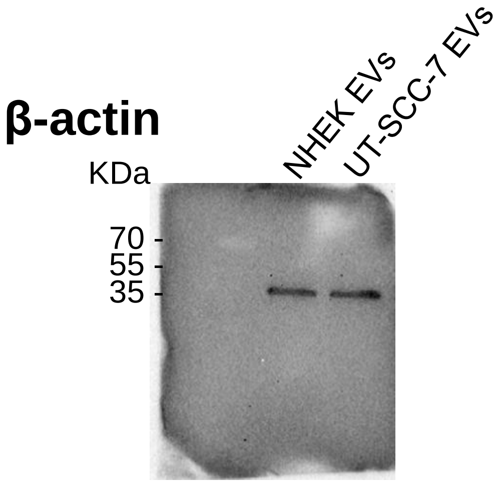

Supplement: Supplementary file 2 — Western blot_uncropped blots [file 41420_2023_1555_MOESM2_ESM.pdf]
